# Immune microenvironment changes of liver cirrhosis: emerging role of mesenchymal stromal cells

**DOI:** 10.3389/fimmu.2023.1204524

**Published:** 2023-07-19

**Authors:** Qiuyun Yi, Jinxian Yang, Ying Wu, Ying Wang, Qiqi Cao, Wen Wen

**Affiliations:** ^1^ National Center for Liver Cancer, Third Affiliated Hospital of Naval Medical University, Shanghai, China; ^2^ International Cooperation Laboratory on Signal Transduction, Third Affiliated Hospital of Naval Medical University (Second Military Medical University), Shanghai, China; ^3^ Department of Breast and Thyroid Surgery, Changhai Hospital, Naval Military Medical University, Shanghai, China; ^4^ Department of Laboratory Diagnosis, Third Affiliated Hospital of Naval Medical University (Second Military Medical University), Shanghai, China

**Keywords:** liver cirrhosis, liver immune microenvironment, mesenchymal stromal cells, nonalcoholic fatty lives disease, autoimmune liver disease, chronic liver disease, therapy

## Abstract

Cirrhosis is a progressive and diffuse liver disease characterized by liver tissue fibrosis and impaired liver function. This condition is brought about by several factors, including chronic hepatitis, hepatic steatosis, alcohol abuse, and other immunological injuries. The pathogenesis of liver cirrhosis is a complex process that involves the interaction of various immune cells and cytokines, which work together to create the hepatic homeostasis imbalance in the liver. Some studies have indicated that alterations in the immune microenvironment of liver cirrhosis are closely linked to the development and prognosis of the disease. The noteworthy function of mesenchymal stem cells and their paracrine secretion lies in their ability to promote the production of cytokines, which in turn enhance the self-repairing capabilities of tissues. The objective of this review is to provide a summary of the alterations in liver homeostasis and to discuss intercellular communication within the organ. Recent research on MSCs is yielding a blueprint for cell typing and biomarker immunoregulation. Hopefully, as MSCs researches continue to progress, novel therapeutic approaches will emerge to address cirrhosis.

## 1 Introduction

**Figure 1:**
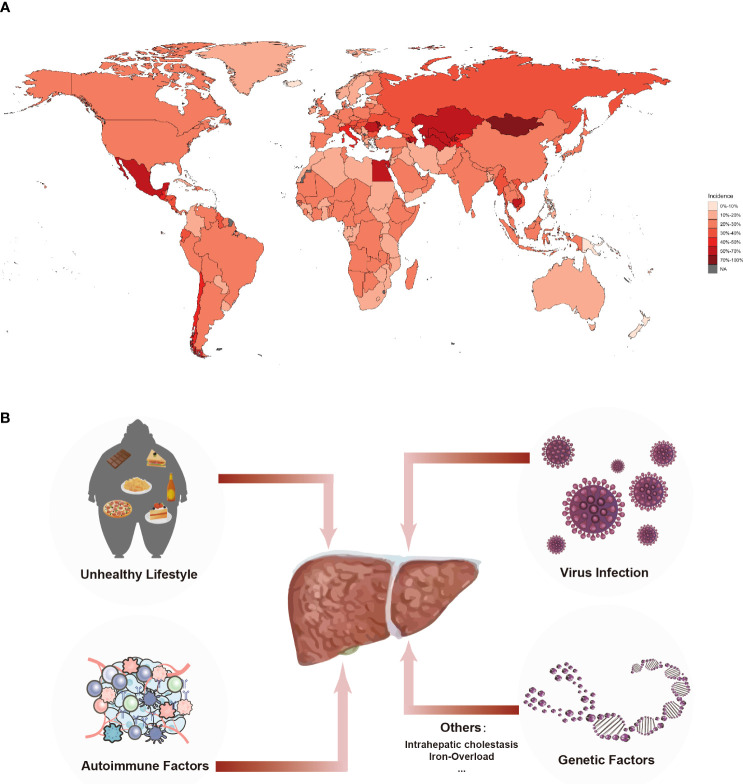
Incidence and Etiology of Liver Cirrhosis. **(A)** Incidence of liver cirrhosis in chronic liver diseases worldwide. Around 1.5 billion people worldwide suffer from chronic liver diseases that eventually progresses into fibrosis and cirrhosis, result in 10 thousand deaths globally. The data were extracted from the GHDx database (https://ghdx.healthdata.org). **(B)** The etiology of cirrhosis. Leading etiologies of cirrhosis were viral, alcoholic, unhealthy lifestyle and genetic factors.

According to epidemiological data (1), approximately 1.5 billion people worldwide suffer from chronic liver disease (CLD), with about 20,000 deaths occurring annually, of which 10,000 are caused by liver cirrhosis. The global mortality for liver cirrhosis has risen by 47.15% in recent years (2, 3). Viral hepatitis, alcoholic liver disease, and non-alcoholic steatohepatitis are the leading causes of liver cirrhosis (4). Moreover, a wide range of other factors also can lead to cirrhosis, including genetic factors, autoimmune diseases, cholestatic diseases, iron or copper overload (5). Hepatitis B virus (HBV) and hepatitis C virus (HCV) are responsible for more than 60% of cirrhotic cases worldwide (6). The number of hospitalized patients with HCV-related cirrhosis is anticipated to decrease significantly by 2025 (7). Only a small number of patients infected with the hepatitis D virus (HDV) will develop liver cirrhosis (8). In pregnant women, low immune function often plays a role as a prerequisite for liver cirrhosis when infected with the hepatitis E virus (HEV), with up to 30% of pregnant patients dying from HEV infection (9). Alcohol-related cirrhosis (AC) has been shown to be a significant cause of hospitalization in the United States, with the number of hospitalized patients increasing rapidly (10). A survey of middle-aged women in the UK found that the higher the amount of alcohol consumed, the greater the incidence of liver cirrhosis (11). Due to the development of hepatitis virus vaccines and effective antiviral therapy, the incidence and prevalence of end-stage liver cirrhosis in non-alcoholic fatty liver disease (NAFLD) change to has risen sharply (12). The prevalence rate of NAFLD-related end-stage liver cirrhosis in China is growing at an alarming rate with the accelerating urbanization process. It is estimated that the number of NAFLD patients in China will reach 314.58 million by 2030 (13). We concluded the epidemiology and risk factors for liver cirrhosis (**Figure 1**).

Cirrhosis is an end-stage pathological process caused by a variety of chronic liver diseases that will result in persistent chronic liver injury (14). Cirrhosis, characterized by chronic inflammatory necrosis and dynamic fibrosis, is considered to be a diffuse pathological state with a transformation from normal liver tissue structure to abnormal nodular hyperplasia, which in turn progresses from compensated cirrhosis (asymptomatic stage) to decompensated cirrhosis (symptomatic stage) (5), eventually leading to hepatocellular carcinoma (15). However, studies have found that this process can be prevented, bringing about reversible liver fibrosis and the reversal of cirrhosis (15, 16). Regardless of the complexity and prevalence of the etiology of cirrhosis, liver fibrosis is a mandatory part of cirrhosis. Chronic inflammatory liver injury and liver fibrosis continue to increase, leading to dysregulated crosstalk between immune cells in the liver microenvironment, which drives the progression of cirrhosis (17–19).

## 2 Liver cirrhosis immune microenvironment

In addition to its role in metabolism, nutrient storage, and detoxification, the liver is the body's most functionally complex immune organ. It has a profound impact on immune function (20). The liver is rich in blood circulation and the circulation system collects blood from the portal vein and the hepatic artery, which contains a large number of microbial-associated molecular patterns (MAMP), pathogen-associated molecular patterns (PAMP), damage-associated molecular patterns (DAMP), and various toxin and antigen molecules from the intestine (19, 21). Herein, the liver must simultaneously recognize antigenic components from the systemic circulation and the gastrointestinal tract. These antigens stimulate the liver through a series of pattern recognition receptors (PRR), such as Toll-like receptors (TLR) and nucleotide-binding oligomeric domain-like receptors (NOD-like receptors or NLR), which trigger unique immune responses to induce immune activation and immunomodulatory cytokine production. TLR is expressed on various hepatic cells, like Kupffer cells (KCs), dendritic cells, hepatic stellate cells, endothelial cells and hepatocytes (22). The hepatic immune microenvironment contains a variety of immune cells and molecules performing unique roles based on the association with non-immune cells, thus developing a complex and dynamic network system. Although the irreplaceable metabolic functions of the liver often obscure the perception of its role as an immune organ, hepatic metabolic functions create a microenvironment in which parenchymal and non-parenchymal cells communicate; in other words, the metabolic environment can alter the immune response in the liver (23).

**Figure 2:**
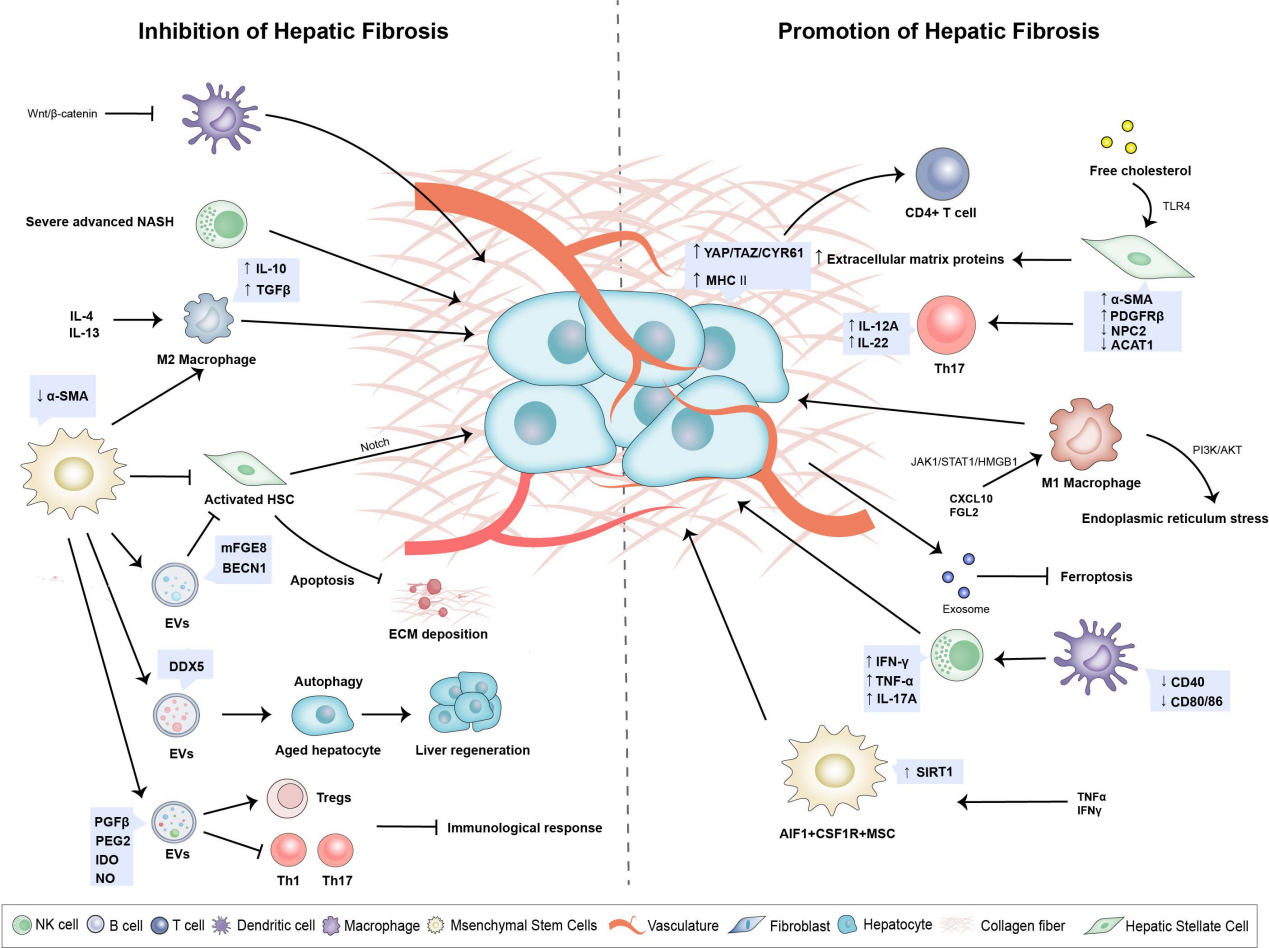
Changes of hepatic immune microenvironment play a pivotal role in hepatic fibrogenesis. A variety of immune cells and non-immune cells constitute a complex and dynamic network system. Upregulation of YAP/TAZ/CYR61 in activated Hepatocytes activating monocyte differentiation into pro-inflammatory macrophages. Activated HSCs promote lipid droplet loss and α-smooth muscle actin increase as well as secretion of extracellular matrix proteins and accelerate the development of liver fibrosis. Hepatic Macrophages promote autophagy and activation of HSC by secreting prostaglandin E2 and binding to receptor EP4, which leads to the development of liver fibrosis and cirrhosis. MSCs can inhibit the inflammation and immune response, inhibit the excessive ECM deposition, and promote the hepatocyte regeneration during liver fibrosis.

The liver microenvironment consists of multiple components, including KCs, hepatic sinusoidal endothelial cells (HSECs), HSCs, immune cells, extracellular matrix (ECM), cytokines, and various growth factors (24, 25). Along with the liver’s inherent immune dysfunction, viral infections, alcohol abuse, metabolic disorders, and autoimmune abnormalities can indirectly inflict liver injury, inflammation, fibrosis, and cirrhosis. Changes to the immune microenvironment in liver cirrhosis involve a decrease in CD8^+^ T cells and natural killer (NK) cells and an increase in CD4^+^ memory T cell infiltration (26) (**Figure 2**).

### 2.1 Hepatocytes

Hepatocytes are involved in the innate immune response by undergoing organelle damage and releasing stress signals in response to injury and inflammatory stimulation, promoting the development of liver cirrhosis and cancer. This process occurs with complicated crosstalk between hepatocytes and immune cells in the liver microenvironment (27, 28). In mice, activated hepatocytes can induce monocytes into pro-inflammatory macrophages with increased YAP/TAZ/CYR61, stimulating liver inflammation and fibrosis (28, 29). YAP/TAZ is the vital effector in the Hippo pathway, which regulates TGF-β2–mediated fibrogenesis (30). MHC-II is highly expressed in hepatocytes of alcoholic hepatitis, and it can activate CD4-positive lymphocytes and trigger a pro-inflammatory response (31). Lipid deposition can increase the susceptibility of hepatocytes to apoptosis in patients with nonalcoholic steatohepatitis (NASH), which had demonstrated in high-fat diet (HFD) mice. Notably, lacking AMP-activated protein kinase (AMPK) can accelerate fibrosis in NASH (32). Virus-infected and hepatocyte-derived exocrine miR-222 promoted fibrosis by inhibiting TFRC and TFRC-induced ferroptosis (33).

The overexpression of transcription factor FoxM1 was dependent on Kupffer cells, and it triggered hepatocyte death and contributed to liver inflammation and injury (34). Hepatocyte autophagy is a steady-state process that protects against hepatocyte death (27, 35). In CCl4-induced mouse models and cirrhotic patients, hepatocyte autophagy was significantly inhibited by the miR-125a/VDR axis-dependent autophagy, which finally promoted liver fibrosis (36). Autophagy disorders were also observed in alcoholic liver disease (ALD) and NAFLD (37). While the situation was different in viral hepatitis. Hepatocyte autophagy could enhance HBV DNA replication (38), while autophagy disorders could inhibit HCV replication by enhancing intracellular immunity (39). Telomere shortening and the absence of telomerase in hepatocytes could lead to cell senescence, promoting virus replication and liver cirrhosis (40, 41).

### 2.2 Hepatic stellate cells

HSCs reside in the Disse space between hepatic sinusoidal endothelial cells (LSECs) and hepatocytes. In their resting state, HSCs contain many retinol (vitamin A) lipid droplets (42). However, when the liver was subjected to inflammatory stimulation or hepatocyte death, HSCs received signals secreted by immune and non-immune cells in the liver microenvironment and underwent transdifferentiation into proliferative fibroblast myofibroblasts (MFs) (43). Activated HSCs lost lipid droplets and upregulated the expression of α-smooth muscle actin (α-SMA) (44), which led to the secretion of extracellular matrix proteins and the eventual development of liver fibrosis (17). The percentage of α-SMA positive hepatic stellate cells was significantly increased in patients with virus-associated cirrhosis (45). HSC activation was driven by the increased level of platelet-derived growth factor (PDGF) receptor β (46). Kupffer cells secreted PDGF, which could stimulate the production and deposition of collagen. Additionally, activation of the acid-sensing ion channel 1a (ASIC1a) via the PI3K/AKT pathway induced endoplasmic reticulum stress (ERS), thereby promoting the progression of liver fibrosis (47, 48). Apart from retinoids, cholesterol, triglycerides, phospholipids, and free fatty acids are also present in HSC lipid drops (49). The NPC2 protein expressed in resting HSC, as well as the ACAT1 isoenzyme, together bound directly to free cholesterol and played a critical role in cholesterol metabolic homeostasis. The accumulation of free cholesterol stimulated HSC through increasing TLR4 signaling and sensitizing HSC to transforming growth factor β (TGF-β) (50, 51). In a high-cholesterol diet mouse model of NASH, NPC2 and ACAT1 deficiency significantly boosted liver fibrosis progression (52, 53).

T helper cells (Th17s) collaborated with HSCs in a pro-inflammatory circumstance. Activated HSCs recruited more Th17 cells and provoked the secretion of IL-12A and IL-22 that contributed to cirrhosis in chronic hepatitis B (CHB) (54). Regulatory T cells (Tregs) possess anti-inflammatory properties. IL-8 produced by Foxp3^+^CD4^+^ Tregs activated HSCs and promoted liver fibrogenesis in chronic hepatitis C (43). 22-carbon hexanoic acid (DHA) plays a critical role in anti-fibrotic activity depending on peroxisome proliferator-activated receptor γ (PPARγ), while it is absent in liver cirrhosis patients, low level of DHA promotes NF-κB and TGF-β pathways in HSC and consecutively activates HSC (55, 56). Additionally, it was found that membrane-bound glycoprotein CD73 promoted activation and autophagy of HSCs by promoting AMPK/AKT/mTOR signaling pathway, which was conducive to alcohol-related liver fibrosis (57).

Cell-derived extracellular vesicles (EVs) have emerged as essential agents in the progression of liver injury and fibrosis (58). Delivering diverse cargo via EVs is a critical component of cell-to-cell communication (59). In the liver, EVs from injured hepatocytes and LSECs activate and migrate of HSCs (1, 58). Recent research shows that SHP2 in HSCs exerts its pro-fibrotic role by enhancing the release of fibrogenic EVs through inhibiting autophagy, REDD1, and activating the mTOR pathway (60).

### 2.3 Mesenchymal stromal cells

Mesenchymal stromal cells (MSCs) are multipotent fibroblast-like cells that have the ability to differentiate into hepatocyte-like cells (HLCs) and immunomodulatory properties have received much attention in a wide range of medical and health fields (61).MSC was reported to express a specific set of surface markers, such as CD73, CD90 and CD105 (62). 

Single-cell RNA sequencing analysis unveiled that different subsets of MSCs were functionally distinct, and even though CMKLR1+ MSCs had lower proliferative capacity than CMKLR1- MSCs, the former had superior immunomodulatory functions (63). In addition, Zong et al. also identified another isoform by using the high-throughput sequencing technology, AIF1+CSF1R+MSCs, with high expression of SIRT1 and induced by TNF-α, exerting pro-inflammatory and pro-tumorigenic effects (64). Similar to HSCs, MSCs are one of the sources of MFs in the liver and are highly differentiated (65). Nevertheless, MSCs could suppress HSC activation and protect hepatocytes from damage by inhibiting the Notch pathway, thus alleviating the progression of liver fibrosis to cirrhosis (66). It has been shown that MSCs only become immunosuppressive when exposed to sufficiently high levels of pro-inflammatory cytokines (67–69). Despite their higher pro-inflammatory potential, MSCs can exhibit pro-inflammatory phenotypes when exposed to low levels of IFN-γ and TNF-α. Through the production of chemokines, they enhance T cell response by bringing lymphocytes to areas of inflammation (67).

MSCs have been shown to exert significant therapeutic effects utilizing their soluble products, such as extracellular vesicles, cytokines, trophic factors, and chemokines. Research shows that the EVs generated by MSCs, such as exosomes, could make great contributions to the therapeutic potential in tissue repair, angiogenesis and immunomodulation by facilitating cell–cell interactions, and delivering paracrine factors (70). MicroRNA-618, the exosome of MSCs, acted as an instrumental player targeting Smad4 to reverse the progression of fibrosis to cirrhosis (71). Exosome-derived Bone marrow stromal cell-derived exosomes (BMSC-Exos) attenuated collagen deposition and liver impairment, enhanced hepatocyte proliferation and ultimately alleviated liver fibrosis in a rabbit cirrhosis model (72). BMSC-Exos suppressed hepatocyte pyroptosis by downregulating pyroptosis-related proteins which included NLRP3, caspase-1, and IL-1β, thereby remitting liver cirrhosis (73).

Moreover, the exosomes secreted by MSC have similar physiological functions as MSCs and play a major role in cellular communication (74, 75). They can also induce anti-inflammatory M2 polarization and facilitate the production of anti-inflammatory mediators such as IL-10 and TGF-β (76). HSC ferroptosis can also be mediated by MSC-exosome (MSC-Exo) to mitigate liver fibrosis (77).

It has been shown that MSCs regulate the innate and adaptive immune response through intercellular contacts or paracrine mechanisms (67). As an illustration, MSCs can produce HGF and IL-6, which inhibit monocyte differentiation into dendritic cells, lowering inflammation, decreasing the secretion of IL-12 and IFN-γ, and increasing the production of IL-10, continually weakening the activation of T cells (78). MSCs inhibit the Kupffer cell activity, reducing the production of the pro-inflammatory cytokine TNF. Furthermore, MSCs secrete PGE2 to transform pro-inflammatory M1 macrophages into anti-inflammatory M2 macrophages (79). MSCs suppress CD8^+^ T lymphocyte proliferation and enhance CD4^+^ T lymphocyte conversion from T-helper 1 to T-helper 2 phenotype by producing IDO and heme oxygenase 1 (80).

Furthermore, recent research suggests that autophagy and senescence are mechanisms through which MSCs acquire their antifibrotic properties. As a vital cellular process, autophagy prevents nutritional, metabolic, and infection-mediated stress while maintaining homeostasis (81). The efficacy of MSCs as a therapeutic intervention is contingent upon the maintenance of optimal levels of autophagy, which in turn can ameliorate the fibrotic cascade. Despite this, aging-related autophagic damage is associated with a decline in MSC number and function, which are crucial to liver fibrosis (82).

### 2.4 Liver sinusoidal endothelial cells

LSECs are non-substantial hepatic endothelial cells lacking basement membranes and rich in open window pores. These LSECs window pores serve as a parclose to protect hepatocytes from various damages and facilitate substance exchange by producing nitric oxide (NO) for stimulating vascular endothelial growth factor (VEGF) and reversing activated HSC to a resting state (83–85). The alcohol-metabolizing enzyme Cytochrome P4502E1 (CYP2E1) was expressed in alcohol-induced LSEC, leading to increased acetylation of mitochondrial heat shock protein 90 (Hsp90). This acetylation reduced the interaction between Hsp90 and nitric oxide synthase (eNOS), resulting in decreased NO production and increased alcohol-induced liver injury (86). Similarly, Notch signaling was activated in LSECs of NASH mice and exacerbated NASH progression in an eNOS-dependent mechanism (87). LSECs possessed endocytic and clearance abilities and vital immune functions, impacting the homeostasis of the liver microenvironment (88, 89). During the early stage of liver cirrhosis, LSECs exhibited anti-inflammatory effects (90). Upon microbial infection, LSECs triggered local activation of effector CD8 T cells that exerted the immune surveillance capacity of the liver (88). Immunoproteasome LMP7 levels in LSECs were elevated in cirrhosis patients and liver fibrosis mice models, and LSECs presented MCH-II antigen to CD4 T cells after liver injury stimulation (91).

Hepatocyte death can lead to LSECs capillarization, immune cell interactions, and HSCs activation (92). Hepatic sinusoidal capillarization is the underlying pathological change of liver cirrhosis (93). This transformation was deleterious in NASH to form a basement membrane on LESCs’ surface, which inhibited the release of very low-density lipoprotein (VLDL) from hepatocytes into the Disse cavity and finally promoted hepatic steatosis. Capillarization also invoked hedgehog (Hh) signaling and exacerbated liver cirrhosis development (94, 95). Adipocyte fatty acid binding protein (A-FABP) regulated lipid metabolism, and elevated expression of A-FABP was observed in cirrhosis-associated NAFLD. Meanwhile, A-FABP stimulated Hh signaling and promoted LSECs vascularization, which led to HSC activation to enhance TGF-β1 activity, resulting in more severe liver fibrosis (96). In the progression of liver fibrosis, CXCR4 and CXCR7 exerted opposite effects on LSECs. With the increase of HIF-1α, CXCR4 upregulated to promote the isoform PDGF-BB secretion by LSEC and binding to its receptors, forming an intercellular crosstalk that activated HSCs and aggravated fibrosis, promoting the development of cirrhosis. While CXCR7 downregulation facilitated the capillarization of LSEC to promote hepatic cirrhosis (97).

### 2.5 Dendritic cells

DCs, as the most important antigen-presenting cells (APC), serve as a bridge between innate and adaptive immunity. DCs recognized and ingested pathogenic antigens through phagocytosis with other immune cells and presenting MHC peptides to CD4 T cells and CD8 T cells to initiate the immune response for exogenous antigens (98). DCs include plasmacytoid DC (pDC) derived from common dendritic progenitor (CDP) cells and the conventional dendritic cell subtype generated by the entry of circulating cDC precursors into the peripheral environment (99).

Comprehensive single-cell RNA sequencing analysis revealed that cDCs were associated with NASH pathology. Elevated Xcr1cDC1 was observed in the NASH model to increase pro-inflammatory CD8T cells and exacerbated NASH to cirrhosis (100). The deficiency of Cbl-b and c-Cbl in DCs led to the excessive accumulation of cDC1 in the liver and promoted liver cirrhosis and premature death in mice (101). The Wnt/β-catenin pathway is crucial in liver homeostasis (102). Lack of Wnt/β-catenin signal should be triggered autoimmune hepatitis (AIH) and abnormal activation of hepatic dendritic cells (HDCs), promoting cholestatic liver injury and fibrosis (103). DCs induced NK cells to proliferate and produced IFN-γ, and DC-NK crosstalk severely impaired the ability of antiviral immune response in CHB patients (104). DCs were rapidly recruited to the liver of NASH mice model with elevated TNF-α, IL-6, and MCP-1 expression. DCs depleting delayed intrahepatic inflammation and fibrosis regression, thereby promoting NASH. Chronic alcohol consumption decreased the production of cytokines such as TNF-α, IFN-γ and IL-12 in DCs, and the number of peripheral blood DCs. It also decreased the expression of CD40, CD80, or CD86, which reduced the stimulatory function of DCs on T cells and led to immune deficiency in mice (105). CCL20, produced primarily by HSCs, is a chemoattractant for immature dendritic cells with inflammatory molecules mediating fibrosis. Interaction between immune cells resulted in the increased expression of CCL20 in NAFLD fibrosis patients (106). Indoleamine 2,3-dioxygenase 1 (IDO1), an immunomodulatory enzyme, was highly expressed in choledochotomy (BDL)-induced mice, inhibiting DCs maturation and T cell proliferation from recruiting immune cells and promoting hepatic fibrosis (107).

### 2.6 Natural killer cells and natural killer T cells

NK cells have a powerful killing function, whose amount and activity are always affected by the liver immune microenvironment. They serve as surveillance to monitor external infections, tumors, inflammatory stimuli, and autoimmunity and secrete cytokines and chemokines (108). NK cells are divided into two subgroups: CD56^dim^ (> 90%) group and CD56^bright^ group. The former has a more substantial cytotoxic effect and plays a role as an immunomodulator (109). NK cells are the first line of defense against viral hepatitis, exerting an antiviral immune response by directly clearing virally infected cells or activating antigen-specific T cells via the production of IFN-γ and TNF-α (110). The decrease of CD56^dim^ NK cells, total NK cells, and their activated receptor NKG2D in peripheral blood monocytes (PBMC) of NAFLD patients can lead to NK cell dysfunction (111, 112). In particular, NK cell function was defective and inactivated in patients with CHB, and monocytes suppressed HBV-specific T cell immune responses, leading to chronic persistent HBV infection (113). The increase of CD56^bright^ NK cells could be detected in patients with autoimmune-mediated liver disease, and elevated serum IFN-γ levels induced hepatocyte death by enhancing the cytotoxicity of NK cells, ultimately resulting in macrophage activation and the development of fibrosis (114).

NKT cells possess NK cell-like characteristics and express T cell receptors, which recognize lipid antigens from major MHC-1-associated protein CD1d (115, 116). Invariant natural killer cells (iNKTs) are the primary subtype of NKT cells (109). Patients with HBV-associated liver cirrhosis (HBV-LC) showed highly activated peripheral iNKT cells, which may lead to overhealing caused by extracellular matrix deposition and the progression from fibrosis to cirrhosis (117). Liver injury induced by concanavalin A (ConA) intravenous administration was considered as an experimental model of T cell-mediated AIH in mice, in which iNKTs were specifically activated to kill hepatocytes and accumulated in the mouse liver, increasing activated immune cells cytokine through upregulation of Fas/FasL in the liver, resulting in more severe immune damage (118). With the prevalence of obesity, excessive cholesterol uptake directly destroys the function of NKT cells through lipid oxidation during the progression of NAFLD disease to liver cirrhosis. Interestingly, NKT cell depletion occurred in the early stage of mild NASH. For severe advanced NASH, NKT cells were protective against disease progression and played an anti-fibrotic role (119, 120). Compared with healthy people, primary biliary cholangitis patients had more iNKT cells, which produced high levels of IL-17A and promoted the progression of PBC-related fibrosis (121).

### 2.7 Macrophage

For the complexity of the liver microenvironment and immune function, macrophages show great plasticity and heterogeneity (122, 123). Macrophages can polarize into M1 cells and M2 cells. The classical M1 subtype is activated by TLR ligand and IFN-γ and secretes pro-inflammatory cytokines. On the contrary, the alternative M2 subtype secretes anti-inflammatory cytokines, which are stimulated by IL-4 or IL-13 (124). In the progression of NAFLD, it was found that hepatic macrophages polarized toward M2 and promoted HSC autophagy and activation by secreting prostaglandin E2 (PGE2) and then binding with EP4, which in turn favored the development of liver fibrosis and cirrhosis (125). It was reported that fibrinogen-like 2 (Fgl2) mediated mitochondrial damage, disrupted mitochondrial HSP90-Akt interactions. Moreover, Fgl2 induced M1 Polarization to secrete pro-inflammatory factors in hepatitis B (126). CXCL10 promoted M1 polarization, resulting in the activation of the JAK/STAT1 pathway (127). The connection between macrophages and HSCs can facilitate liver fibrogenesis. Subtype M2C-like polarized macrophages activated tyrosine kinase receptors (MerTK) on their surface influencing the profibrogenic HSCs (128).

M1 Polarization of macrophages is critical in the liver. Xu et al. found that osteopontin promoted M1 Polarization in NAFLD by activating JAK1/STAT1/HMGB1 signaling, which aggravated liver injury and cirrhosis (129). Studies in a humanized mouse model of HBV infection revealed that HBV could induce the differentiation of human monocytes/macrophages into M2 macrophages, which then expressed IL-10 and other inhibitory cytokines (113, 130). In addition, soluble CD206 (sCD206) expressed by macrophages could promote T cell activity and inhibit the antiviral effect of CD8T cells. High expression of sCD206 accelerated the progression of cirrhosis in patients with hepatitis B virus-related decompensated cirrhosis (HBV-DeCi) (131).

## 3 Therapies

Treatment of the etiology is the cornerstone of cirrhosis treatment. The means of treating the primary cause include alcohol abstinence for alcoholic cirrhosis, antiviral drugs for HBV and HCV, immunosuppressants for autoimmune hepatitis, and ursodeoxycholic acid for primary biliary cholangitis. Several studies have shown that etiologic treatment can effectively restrain the progression of cirrhosis and even reverse patients with decompensated cirrhosis to compensated cirrhosis (i.e., recompensated cirrhosis), thus reducing the rate of death and improving the quality of life. Therefore, better studies of the altered cirrhosis immune microenvironment would help to develop more effective targeted therapeutic regimens.

### 3.1 Viral hepatitis

Currently approved antiviral therapies for HBV include pegylated interferon alpha (PEG-IFN-α) with immunomodulatory activity and nucleoside (acid) analogs (NAs) that inhibit HBV polymerase. Still, neither achieves the functional cure of HBV (i.e., scavenging HBsAg) (132). The conditions for the usage of the two drugs are different. NAs can prevent severe viral hepatitis relapse, especially in patients with liver cirrhosis. While PEG-IFN-α is contraindicated in cirrhosis patients, for it can cause more severe liver damage (133). In a randomized open phase II trial, treatment with elbasvir/grazoprevir (EBR/GZR) + sofosbuvir (SOF) for 12 weeks was highly effective for HCV patients treated either with or without peginterferon (PEG-IFN-α-2a) or for cirrhosis patients (134).

The most advanced approach in clinical development to date is the competitive inhibitor myrcludex-B (MyrB) based on the PreS1 peptide now called the hepatocyte entry inhibitor bulevirtide (BLV), which has successfully blocked HBV and HDV entry (135). In HDV-associated cirrhosis patients, for whom interferon is contraindicated, treatment with BLV alone results in a sustained virologic response (136), but the optimal duration remains determined (137). The combination of BLV and tenofovir disoproxil fumarate (TDF) has a favorable safety and efficacy profile for treating HDV-related compensated cirrhosis (138). Lonafarnib (LNF) and ritonavir (RTV) are promising therapies for treating HDV, and the combination of PEG-IFN-α may increase the efficacy. A phase III clinical trial of LNF is currently underway (139).

The risk of HCV progression to cirrhosis and HCC continues to increase after treatment with direct-acting antiviral agents (DAAs). Dysfunction of CD4^+^ and CD8^+^ T cells has been identified in patients with hepatitis C, making liver immunotherapy urgent (140). Compared with an oral agonist of TLR 7 (GS-9620), the agonist of TLR 8 (GS-9688) stimulated the expression of IFN-γ and TNF-α in NK cells while all increased the antiviral capacity of CD8^+^ T cells (141). The immunotherapy by GS-9688 achieved sustained efficacy in murine models of HBV (142). The safety and tolerability of oral selgantolimod (TLR 8 agonist) was evaluated in CHB patients in one phase Ib study, and the recent phase II study further supported the development of this immunomodulator (143). NASVAC, a vaccine formulation containing both hepatitis B surface antigen (HBsAg) and hepatitis B core antigen (HBcAg), targeted a lower proportion of patients who developed cirrhosis in phase III clinical trials compared with PEG-IFN (144). HCV vaccine in phase I-II clinical trials found that 78% of HCV-infected patients showed a specific response to T cells, reducing the peak of HCV RNA level, providing a basis for future immunotherapy (145). In a proof-of-concept clinical trial, combination therapy of entecavir (NA) plus PEG-IFN-α-2a followed by HBV vaccination developed a “blueprint” for serum clearance of HBsAg, suggesting that the combination of drugs and immunotherapy provides therapeutic interventions for functional cure of viral infections (146).

### 3.2 Alcoholic hepatitis

Campaigns for vaccination, screening, and antiviral treatment of hepatitis B and C have reduced the burden of chronic disease. However, concurrent increases in drug injection, alcohol abuse, and metabolic syndrome threaten these trends (1). A large randomized clinical trial discovered that long-term administration of albumin could improve survival in patients with decompensated cirrhosis (147). Whereas, in an open-label multicenter trial ATTIRE, increasing albumin infusion in patients with decompensated cirrhosis showed no more benefit due to most of the patients suffering alcohol-related liver disease (148). These results show that breaking down the etiology of cirrhosis is crucial for subsequent treatment.

Corticosteroids are currently recommended for the treatment of severe alcoholic hepatitis (SAH), but about 25% of SAH did not respond to prednisone treatment (149). Granulocyte colony-stimulating factor (G-CSF) can prolong the survival of alcoholic hepatitis (AH) patients, and the combination of N-acetylcysteine (NAC) with standard drug therapy (pentoxifylline) may also reduce AH liver injury and prolong survival (150). The immunomodulatory effect of G-CSF in the AH mouse model had shown to increase the number of immune cells entering the liver and promoting the polarization of macrophages toward M2, which facilitated liver repair (151). Macrophage and neutrophil infiltration diminished in AH mice treated with intraperitoneal MSC infusion, and skeletal muscle satellite cell-derived MSC counteracted ethanol-induced inflammation by secreting PEG2 and HGF, thus making MSC promising as an effective therapy for patients with alcoholic hepatitis (152, 153). In SAH patients, the fecal microbiome transplantation (FMT) treatment for 90 days could cause a reduction in the ratio of mucosa-associated invariant T and Th17 cells and a decrease in IL-17 and IFN-γ production. Besides, FMT could attenuate the hepatic inflammatory response and finally improve survival in SAH patients, which suggests that FMT may be an alternative to prednisone treatment (154). In the Defeat Alcoholic Steatohepatitis trial (DASH), the combination of IL-1β-receptor antagonist (anakinra) with pentoxifylline plus zinc supplementation offered the opportunity for prolonged survival in patients with AH (155). Significantly elevated markers of immune cell activation stimulated by DAMP and PAMP, including macrophages and neutrophils were found in AH subjects studied in four clinical centers in the United States and correlated with the severity of AH (156). Silybin, a kind of herbal plant, normalized alcohol-induced immune regulation of the liver and induced activation of T cells and downregulation of cytokines such as TNF (157). These immune cells play an irreplaceable role in the intricate liver immune microenvironment, which guides the direction for AH to discover immune targets and provides future treatment strategies to prevent disease progression, but further prospective clinical studies are needed to confirm this good desire.

### 3.3 Nonalcoholic fatty liver disease

Anti-inflammatory and anti-fibrotic immunotherapy strategies play a therapeutic role for NAFLD, with no standard treatments change to currently approved (158). FXR is expressed in immune cells. Cilofexor, an FXR agonist, improved hepatic steatosis in a 24-week phase II study in NASH patients. However, Liver Fibrosis scores and liver stiffness were not observed when were used only in noncirrhotic NASH patients (159). Meanwhile, a modified and optimized FXR agonist (MET409) had the same effect in another 12-week study in patients with NASH, despite the side effect of headache. MET409 is intended to be used as first-line monotherapy for NASH, while combinations of MET409 with other agents are in development (160). More recently, results of a phase II trial showed that the FXR agonist tropifexor led to sustained reductions in ALT and liver fat, but the side effect of pruritus was unavoidable, and further investigation for antifibrotic effects in combination with other agents is needed (161). NAFLD is associated with hypertriglyceridemia. In patients receiving the FXR agonist cilofexor (CILO) and the acetyl-coenzyme A carboxylase (ACAC) inhibitor firsocostat, inhibition of triglycerides by fenofibrate was strengthened (162), providing strong evidence for the combinations of multiple drugs. Saroglitazar is a PPAR-α/γ agonist that is no less effective than fenofibrate (163). Whether it can replace fenofibrate in combination therapy for NAFLD remains to be proven.

The immunotherapy based on chimeric antigen receptor (CAR) T cells targeting and destroying myofibroblasts can be used to reduce extracellular matrix deposition in NASH mice (164). But more evidence is needed to confirm the efficacy. Cenicriviroc (CVC), as a dual CCR2 and CCR5 antagonist, had confirmed in a phase IIb (CENTAUR) study in anti-fibrosis effect in NAFLD patients (165). Unfortunately, the CVC clinical trial was prematurely interrupted in the anticipated phase III (AURORA) study (166). Oral OKT3 (anti-CD3 mouse monoclonal antibody) was administered to NASH patients with diabetes to induce Tregs activation for ameliorating insulin resistance and liver injury. Although the number of study subjects was small, the related parameters showed promising results (167). Fibroblast growth factor 19 (FGF19) analog aldafermin (also known as NGM282 or M70) reduced liver fat content and fibrosis levels by 7.7% and 1%, respectively, compared with the placebo group (168). Pegbelfermin is a pegylated FGF21 analog. FGF21 improves the condition of NAFLD and NASH by directly regulating lipid metabolism and reducing fat accumulation in an insulin-independent manner (169). Some clinical trials have shown that BMS-986036 has a good effect on improving the liver fat content, inflammation, and fibrosis of NAFLD and NASH, and is well tolerated (170, 171). As new immunotherapeutic drugs, the clinical research of FGF19 and FGF21 is in the third stage, and more studies are needed to determine their biological characteristics and therapeutic effects (172).

Given that none of the new drugs currently under evaluation shows improvements in major clinical endpoints, it is expected that a reasonable combination will be more effective in controlling or preventing further deterioration of NASH. Several studies have now shown that MSC-secreted exosomes supply natural drug delivery vectors and offer prospective strategies for the treatment of NAFLD. MSC-Exo miR-24-3p, miR-223-3p, and miR-627-5p attenuated lipid deposition and liver fibrosis in NAFLD mice, but more studies are needed to provide meaningful evidence for clinical treatment (173–176). MSC-Exo extracted from human umbilical cords stimulated M2 polarization, exhibited downregulation of pro-inflammatory factors such as TNF-α, IL-6 and IL-1β, and detected high expression of PPARα in liver tissue, thereby alleviating methionine-cholesterol deficient (MCD) diet-induced progression to NASH in NAFLD mice (177). Exosomes originated from human adipose mesenchymal stem cells (hADMSCs-Exo) were shown to inhibit HSCs activation and rectify choline metabolism disorders via PI3K/Akt/mTOR pathway to ameliorate liver fibrosis, especially caused by NAFLD (178). Deeper exploration of immune-related mechanisms and further clinical trials may be needed to cure NAFLD patients.

### 3.4 Autoimmune liver disease

#### 3.4.1 Primary biliary cholangitis

Primary biliary cholangitis (formerly called primary biliary cirrhosis) is characterized by cholestasis and biliary fibrosis, with autoimmune destruction leading to immune-mediated damage (179). Ursodeoxycholic acid (UDCA) is used as first-line therapy for PBC or as second-line therapy (obectocholic acid and benzofibrate) if the patient does not respond to UDCA (180). In a phase III clinical trial, patients who did not respond to UDCA could also be treated with budesonide, which improved liver function markers in serum but had little effect on liver histology (181).

It is well recognized that PPARs are nuclear receptors that regulate a variety of immune cell functions and play an important role in regulating innate and adaptive immunity (182). During the 52-week study, seladelpar, a selective PPARγ-δ agonist, was safe and well tolerated in patients with PBC, which overcame the side effects of skin itching and caused no serious adverse events or deaths (183). Fatigue is also one of the symptoms of PBC. RITPBC is believed to be the first randomized, controlled phase II clinical trial to investigate fatigue in PBC. Rituximab is a monoclonal antibody against CD20 on the surface of B cells to improve serum alkaline phosphatase (ALP) of refractory UDCA in PBC and decimate B cells (184). Rituximab is also considered a potential treatment for PBC fatigue (185). In addition, the anti-IL12/23 monoclonal antibody ustekinumab led to a slight decrease in PBC in a proof-of-concept study, but the efficacy and safety of its immunomodulatory effect remain to be verified (186).

#### 3.4.2 Primary sclerosing cholangitis

Primary sclerosing cholangitis is a chronic cholestatic liver disease characterized by progressive inflammation and fibrosis of the intrahepatic and extrahepatic bile ducts, leading to multifocal biliary stricture and progressive liver disease (e.g., cirrhosis) (187). Immunotherapy for PSC is complicated. Although a variety of immunomodulators have been tested for the treatment of PSC, the general treatment regimen has not been proven to benefit patients (188). 24-Norursodoxycholic acid (norUDCA), a homolog of UDCA, is a novel bile acid that reduces ALP in PSC patients in a dose-dependent way and significantly improves cholestasis (189). According to a meta-analysis, immunosuppressants (mycophenolate mofetil, methotrexate, and tacrolimus) significantly reduced ALP and AST and improved liver function, which seemed to be the most effective treatment with severe side effects (188).

Liver transplantation (LT) is the only life-prolonging and curable treatment for PSC. However, an international observational study found that morbidity and mortality of recurrent primary sclerosing cholangitis (rPSC) increased after liver transplantation in children. It was thought that rPSC elicited a more robust immune response than PSC (190). In addition, a 36-year-old woman who had both PSC and ulcerative colitis was diagnosed with autoimmune hepatitis after treatment with the mRNA vaccine COVID-19 (191). We could speculate whether PSC or a specific immune response after vaccination led to the conversion of immune disease, but the specific mechanism was unclear. Proinflammatory cytokines, such as TNF-α and IL-1β, were highly expressed in patients with PSC and AIH, while the function of T lymphocytes and NK cells in the liver were impaired (192), so anti-TNF therapy was also one treatment option. Exploring the immunological changes in the liver microenvironment may provide a solid basis to clinical immunotherapy.

#### 3.4.3 Autoimmune hepatitis

Autoimmune hepatitis is characterized by elevated serum aminotransferase, immunoglobulin G (IgG) levels, and positive autoantibodies (193). The International Autoimmune Hepatitis (IAIHG) defined “complete biochemical response” (CBR) as the normalization of serum transaminases and IgG (194). To achieve this goal, corticosteroids and/or azathioprine (AZA) are the standard treatment for AIH, but some patients still respond poorly to standard treatment. The CBR rate of patients treated with mycophenolate mofetil (MMF) was significantly higher than that of patients treated with AZA, so MMF became an alternative therapy for initial treatment (195–197). For children with AIH, MMF was a “life-saving drug” for children (198). In most patients with conventionally treated refractory AIH, one in three patients treated with the immunosuppressant calcium phosphatase inhibitor tacrolimus developed CBR and had good renal function after the withdrawal of the drug (199). At the same disease stage, selective depletion of B cells by rituximab, an anti-CD20 monoclonal antibody, also lowered transaminase and IgG levels (200, 201). In a multicenter study, liver stiffness was reduced compared with that before tacrolimus treatment, but no statistical significance was found, possibly due to a small sample size. It is worth noting that only one patient discontinued treatment due to serious adverse events (202). Future studies need to enlarge the number of patients and investigate the effects of immunomodulation on disease.

Depletion of Tregs was one of the methods to establish the AIH mouse model. In conjunction with the reduction of Tregs by steroid treatment, enhanced intrahepatic Tregs immunotherapy would be the preferred option for AIH patients (203, 204). The low dose of IL-2 improves the selectivity and number of Tregs after treatment, thereby re-regulating the hepatic immune microenvironment for improve AIH (204, 205).

### 3.5 Inherited disease

Wilson disease (WD) is an inherited disorder of copper metabolism caused by mutations in ATP7B (hepatomegaly protein) (194). It causes liver damage and neurological symptoms due to abnormal copper ion metabolism in the body, leading to copper accumulation in the liver, brain, and other tissues, which can lead to cirrhosis in the long term (195, 196). Although liver transplantation can cure WD, can also cause serious immunosuppression (197). In most WD patients, oral chelating agents such as D-penicillamine and trientine were effective (206). Trientine tetrahydrochloride (TETA4) was found to be superior to penicillamine in phase III clinical trials (199). Besides, these drugs can decrease the number of whole blood cells in patients, weakening the immune system and increasing the risk of infection (200). Therefore, targeted liver immunotherapy would provide a better cure for WD.

### 3.6 New strategies for MSC based therapy

The present study shows that a number of factors are upregulated by MSCs, such as MMPs and VEGF, to promote liver fibrosis regression (207). Besides, the study indicates A combination of MSCs and macrophages was more effective in reducing fibrotic gene expression and procollagen synthesis than either of them individually. In line with this, it has been observed that localized MSCs improve liver fibrosis by reducing the activation of fibroblasts and the production of collagen (208).

Evidence is mounting that MSC-mediated immune regulation can alleviate liver fibrosis through programmed death mechanisms, such as apoptosis, autophagy, ferroptosis, and pyroptosis (209). Maintaining the characteristics of MSCs is dependent on basal autophagy levels. Aged MSCs can benefit from activated or increased autophagy to slow metabolism and strengthen their functions to resist aging. Conversely, Aged MSCs are more susceptible to toxic substance accumulation and mitochondrial damage, exacerbating inflammatory responses and cell damage, and ultimately this accelerates the aging process. Increasing evidence suggests that fibrotic microenvironment induced MSC autophagy by upregulating Becn1, while Becn1 knockdown inhibited T lymphocyte infiltration, HSC proliferation and suppressed the production of cytokines by increasing PTGS2/PGE2 secretion, thereby further enhancing MSC antifibrotic activity (210). Therefore, the augmentation of the antifibrotic potential of MSCs through the manipulation of their autophagic processes presents a viable approach towards the management of liver fibrosis.

Recently, Zhang et al have proposed the therapeutic potential of extracellular vesicles derived from mesenchymal stromal cells (MSC-Evs) in facilitating liver regeneration (211). MSC-EVs have been observed to stimulate the rejuvenation of aged hepatocytes and augment their proliferation by upregulating mitophagy. Subsequent to a more in-depth inquiry into the mechanistic intricacies of this process, it was discovered that DDX5, which is abundant in MSC-EVs, can be transferred to aged hepatocytes to stimulate EF1 nuclear translocation and consequent upregulation of Atg4B expression, ultimately leading to the induction of mitophagy. The validity of these findings was confirmed through in vivo and in vitro experiments involving DDX5 knockdown in MSC-EVs. Therefore, MSC-EVs present a promising therapeutic modality for liver fibrosis patients by reversing senescence and promoting the regeneration of senescent hepatocytes (211).

## 4 Summary

The disruption of hepatic homeostasis may lead to a persistent inflammatory response and a reduction in immune function, thereby emphasizing the critical role of the immune microenvironment in the development of liver cirrhosis. Remarkably, recent clinical trials have demonstrated that transplantation of mesenchymal stromal cells derived from human umbilical cord blood can potentially improve the long-term survival of patients with decompensated cirrhosis (202). Moreover, the paracrine intercellular communication of MSCs may confer the benefit of effectively eliciting physiological effects through minute concentrations of EV. The diversity of MSCs derived from distinct tissue sources and the distinctive attributes of MSC-EVs indicate a significant therapeutic potential of MSCs for their antifibrotic properties. Nevertheless, the capacity of MSCs to proliferate indefinitely is limited, and they may exhibit senescence after cell division, which can disrupt homeostasis in vivo through senescent cells and cell-cell interactions. Hence, investigating the mechanisms of cellular communication in the aging microenvironment is a pressing necessity and a promising therapeutic strategy.

## Author contributions

QY searched the literature and drafted the manuscript, Ywa and QC collected and sorted out the literature. JY made the figures, QY and JY designed the article structure, and Ywu revised the manuscript. All authors contributed to the article and approved the submitted version.
